# Supplementary material for: Soluble stroma‐related biomarkers of pancreatic cancer
Source: EMBO Mol Med. 2018 Jun 25;10(8):e8741. doi: 10.15252/emmm.201708741 (PMC6079536; doi:10.15252/emmm.201708741)
Supplement: Supplementary file 1 — Appendix [file EMMM-10-e8741-s001.pdf]

Soluble stroma-related biomarkers of pancreatic cancer

Andrea Resovi, Mariarosa Bani, Luca Porcu, Alessia Anastasia, Lucia Minoli, Paola Allavena, Paola Cappello, Francesco Novelli, Aldo Scarpa, Eugenio Morandi, Anna Falanga, Valter Torri, Giulia Taraboletti, Dorina Belotti, Raffaella Giavazzi.

Contents:

Appendix Figure S1 **Page 2**

Appendix Figure S2 **Page 3**

Appendix Figure S3 **Page 4**

Appendix Figure S4 **Page 5**

Appendix Table S1 **Page 6**

Appendix Table S2 **Page 7**

Appendix Table S3 **Page 8**

Appendix Table S4 **Page 9**

Appendix Table S5 **Page 10**

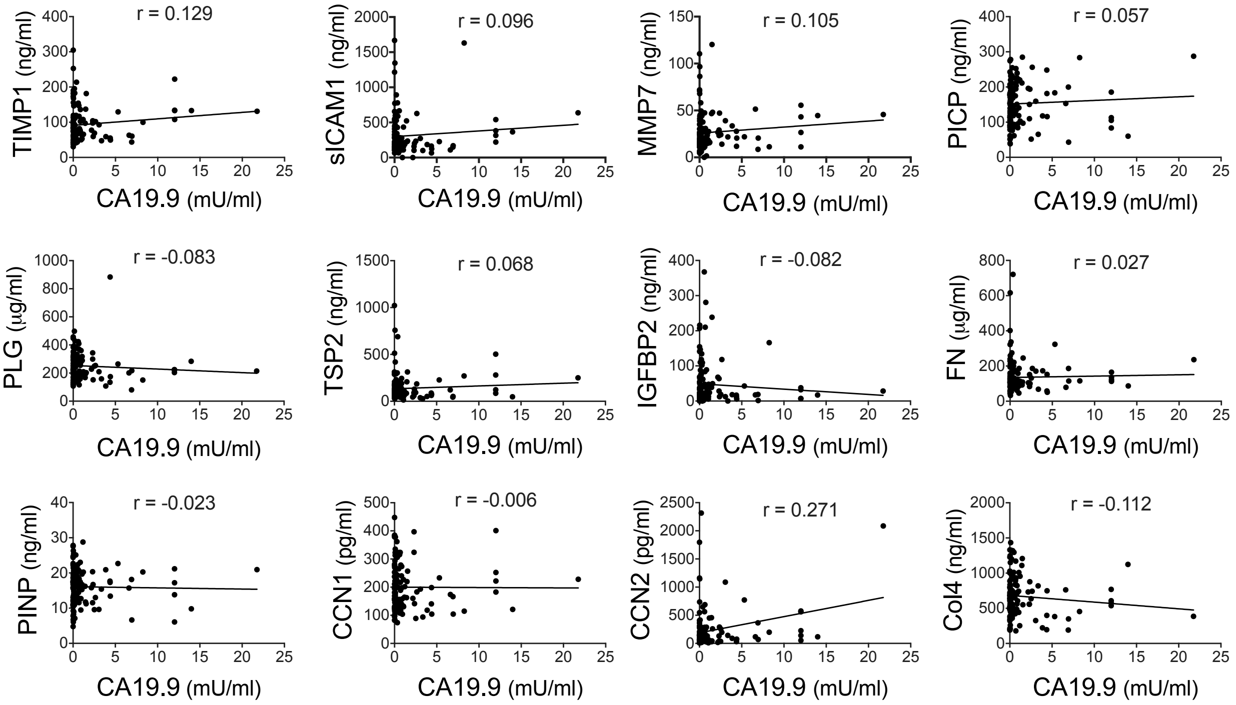

Appendix Figure S1. Lack of correlation between plasma levels of biomarkers and CA19.9 in PDAC patients (second cohort). Pearson correlation coefficients (r).

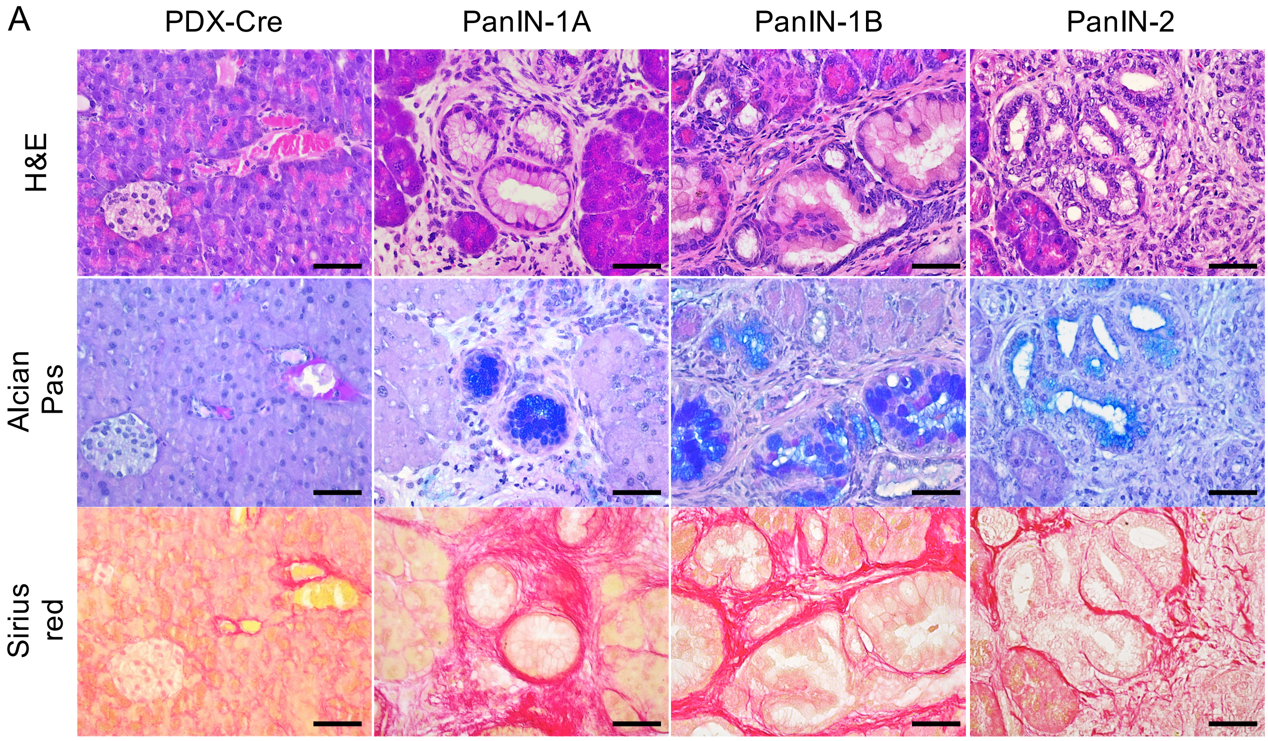

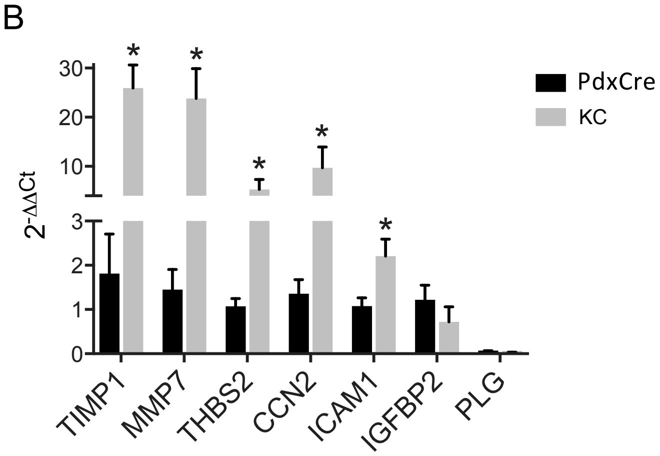

C

Stroma-related molecules	Pdx-Cre vs KC (p-value)
TIMP1	0.0025
MMP7	0.0025
THBS2	0.0061
CCN2	0.0167
ICAM1	0.0242
IGFBP2	0.4121
PLG	0.5273

Appendix Figure S2. A) Histological analysis of pancreas from PdxCre and KC mice with different grades of PanIN lesions at 330 days of age. Hematoxylin-eosin, Alcian pas and Sirius red staining (400X, scale bars: 50µm). B) Expression level of TIMP1, MMP7, THBS2, CCN2, ICAM1, IGFBP2 and PLG analyzed in pancreas from KC mice by RT-PCR. The expression level of target genes was normalized to the geometric median of β-actin and GAPDH housekeeping genes and expressed as $2^{-\Delta\Delta CT}$ (mean ± SEM; Pdx-Cre n=7, KC n=4), *p<0.05 (Mann-Whitney, exact p-values in table C).

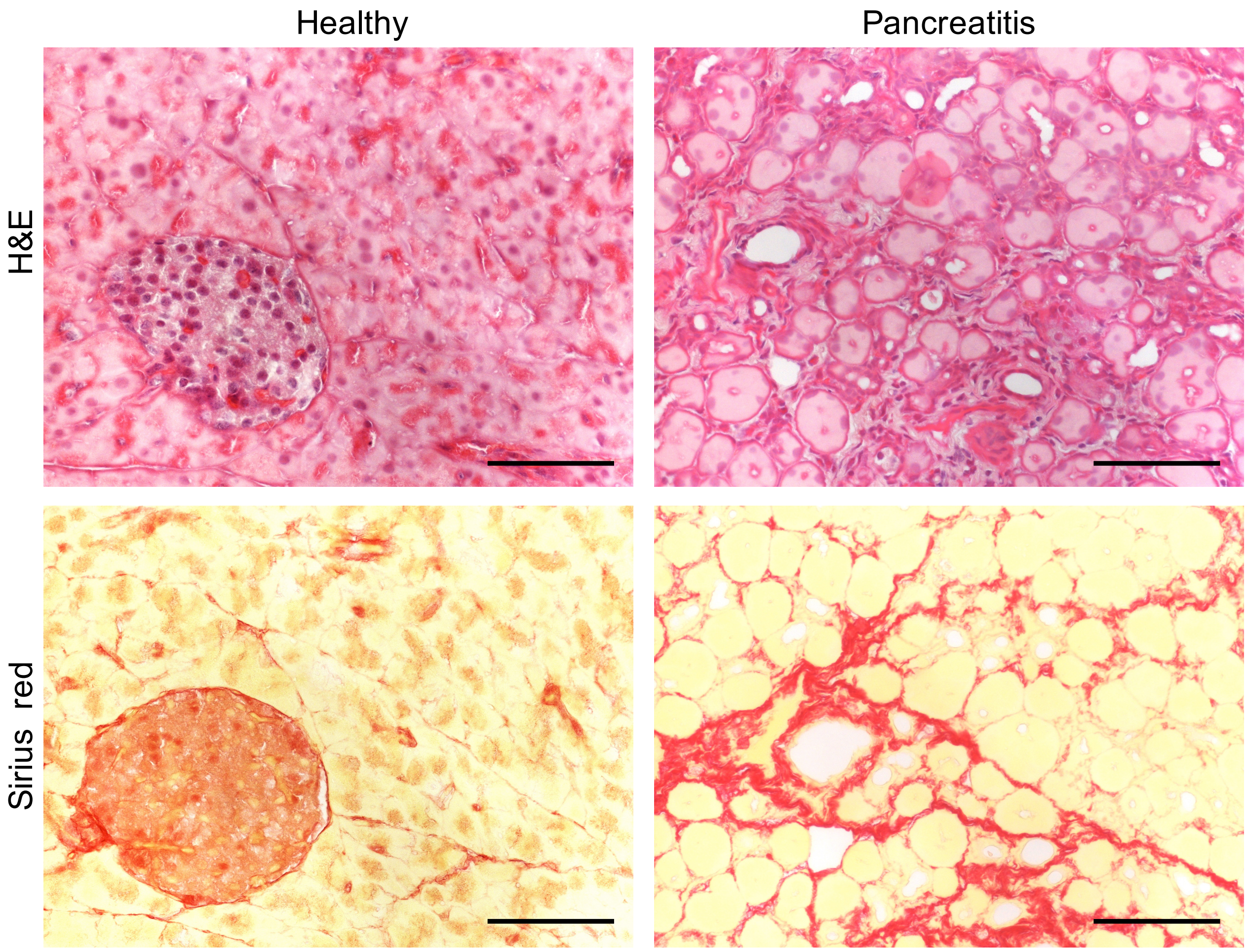

Appendix Figure S3. Histological analysis of pancreas of mice with caerulein-induced chronic pancreatitis. Chronic pancreatitis was induced in C57BL/6 mice by injecting 50 µg/kg of caerulein intraperitoneally six times over five consecutive hours, three times a week for seven weeks. Hematoxylin-eosin and Sirius red staining of pancreas from healthy mice and mice with chronic pancreatitis (400X, scale bars: 50µm). All mice treated with caerulein displayed the typical acinar cell damage associated with the extensive fibrosis of a pancreas with chronic pancreatitis.

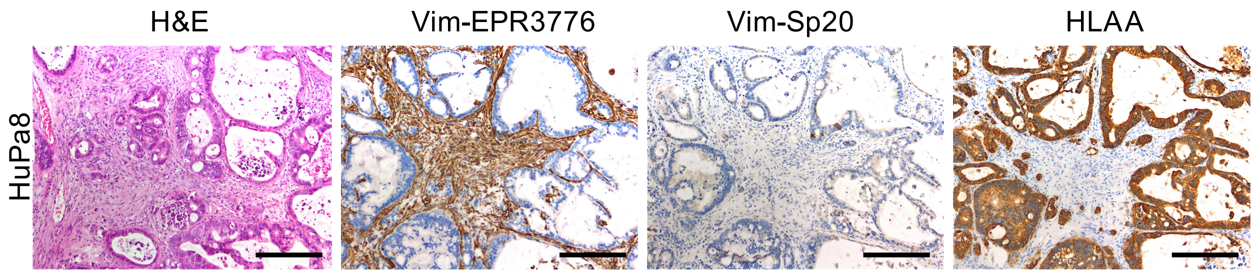

Appendix Figure S4. Hematoxylin-eosin, anti-human and murine vimentin (EPR3776), anti-human vimentin (Sp20) and anti-human HLA A staining of a representative PDAC-PDX (HuPa8). (200X, scale bars: 100µm).

Appendix Table S1. Multiplex and ELISA kits

HUMAN ANALYTES		COMPANY	CATALOG #	TYPE	SENSITIVITY
α2M	alpha-2-Macroglobulin	Millipore	HCVD3MAG-67K	Multiplex	182 pg/ml
CA19.9	Carbohydrate Antigen 19.9	Millipore	HCCBP1MAG-58K	Multiplex	0.3 U/ml
CCN1/Cyr-61	Cysteine-rich angiogenic inducer 61	R & D Systems	DCYR10	ELISA	1.54 pg/ml
CCN2/CTGF	Connective tissue growth factor	CUSABIO	CSB-E07875h	ELISA	1.875 pg/ml
CCN3	Nephroblastoma overexpressed	CUSABIO	CSB-EL015956HU	ELISA	1.95 ng/ml
Col4	Collagen 4	CUSABIO	CSB-E17116h	ELISA	15.6 pg/ml
CXCL7/NAP2	Chemokine (C-X-C motif) Ligand 7	Millipore	HCYP3MAG-63K	Multiplex	11.2 pg/ml
ES	Endostatin	R & D Systems	LAN000	Multiplex	12.8 pg/ml
FGF-2	Fibroblast Growth Factor-2	R & D Systems	LAN000	Multiplex	1.82 pg/ml
FN	Fibronectin	abcam	ab108848	ELISA	700 ng/ml
IGFBP2	Insulin-like Growth Factor-Binding Protein 2	Millipore	HIGFBMAG-53K	Multiplex	1.09 ng/ml
IGFBP4	Insulin-like Growth Factor-Binding Protein 4	Millipore	HIGFBMAG-53K	Multiplex	0.96 ng/ml
IGFBP5	Insulin-like Growth Factor-Binding Protein 5	Millipore	HIGFBMAG-53K	Multiplex	9.87 ng/ml
Lam-P1	Laminin Proteolitic Fragment 1	CUSABIO	CSB-EL027996HU	ELISA	0.47 mU/ml
MMP12	Matrix Metalloproteinase 12	Millipore	HMMP1MAG-55K	Multiplex	82.3 pg/ml
MMP13	Matrix Metalloproteinase 13	Millipore	HMMP1MAG-55K	Multiplex	32.2 pg/ml
MMP2	Matrix Metalloproteinase 2	Millipore	HMMP2MAG-55K	Multiplex	260 pg/ml
MMP3	Matrix Metalloproteinase 3	Millipore	HMMP1MAG-55K	Multiplex	122.9 pg/ml
MMP7	Matrix Metalloproteinase 7	Millipore	HMMP2MAG-55K	Multiplex	167.4 pg/ml
MMP9	Matrix Metalloproteinase 9	Millipore	HMMP2MAG-55K	Multiplex	4.4 pg/ml
NGAL/Lipocalin2	Neutrophil Gelatinase-Associated Lipocalin	Millipore	HSP3MAG-63K	Multiplex	15 pg/ml
PDGF-BB	Platelet-Derived Growth Factor Subunit B	R & D Systems	LAN000	Multiplex	6.87 pg/ml
PEDF/SERPIN	Pigment Epithelium-Derived Factor	Millipore	HNDG2MAG-36K	Multiplex	16 pg/ml
PF4	Platelet Factor 4	Millipore	HCVD3MAG-67K	Multiplex	12 pg/ml
PICP	C-Terminal propeptide of Procollagen 1	CUSABIO	CSB-E08079h	ELISA	15.6 pg/ml
PINP	N-Terminal propeptide of Procollagen 1	CUSABIO	CSB-E11226h	ELISA	4.68 pg/ml
PLG	Plasminogen	CUSABIO	CSB-E09177h	ELISA	15.6 ng/ml
sICAM1	Soluble Intercellular Adhesion Molecule 1	Millipore	HCVD2MAG-67K	Multiplex	39 pg/ml
SPARC	Secreted Protein Acidic and Rich in Cysteine	R & D Systems	DSP00	ELISA	99 pg/ml
sVCAM1	Soluble Vascular Cell Adhesion Molecule 1	Millipore	HCVD2MAG-67K	Multiplex	48 pg/ml
TGF-β1	Trasforming Growth Factor Beta 1	Millipore	TGFB-64K	Multiplex	12 pg/ml
TGF-β2	Trasforming Growth Factor Beta 2	Millipore	TGFB-64K	Multiplex	6 pg/ml
TGF-β3	Trasforming Growth Factor Beta 3	Millipore	TGFB-64K	Multiplex	6 pg/ml
TIMP1	Metallopeptidase Inhibitor 1	Millipore	HTMP1MAG-54K	Multiplex	13.6 pg/ml
TIMP2	Metallopeptidase Inhibitor 2	Millipore	HTMP1MAG-54K	Multiplex	37.4 pg/ml
TSP1	Thrombospondin 1	Millipore	HSP3MAG-63K	Multiplex	133.4 pg/ml
TSP2	Thrombospondin 2	R & D Systems	LAN000	Multiplex	5.48 pg/ml
VEGFA	Vascular Endothelial Growth Factor A	R & D Systems	LAN000	Multiplex	0.81 pg/ml
VTN	Vitronectin	Millipore	HCCBP2MAG-58K	Multiplex	21 pg/ml
MURINE ANALYTES		COMPANY	CATALOG #	TYPE	SENSITIVITY
MMP7	Matrix Metalloproteinase 7	CUSABIO	CSB-E07409m	ELISA	7.81 pg/ml
TIMP1	Metallopeptidase Inhibitor 1	Millipore	MECY2MAG-73K	Multiplex	4 pg/ml
TSP2	Thrombospondin 2	CUSABIO	CSB-EL023488MO	ELISA	125 pg/ml

Appendix Table S2. Heterogeneity and AUC by stage of the molecules analyzed in the confirmatory phase.

Stroma- related molecules	PDAC vs Healthy						
	Odds Ratio				P-value for an interaction between stages	AUC (99%CI)	
	Stage IA-IB-IIA		Stage IIB				
	Point estimate	99%CI	Point estimate	99%CI		Stage IA-IB-IIA	Stage IIB
TIMP1	1.08	1.03-1.13	1.07	1.04-1.11	0.932	0.78 (0.60-0.97)	0.81 (0.72-0.89)
sICAM1 ^	1.08	1.02-1.14	1.09	1.04-1.14	0.723	0.77 (0.60-0.94)	0.77 (0.68-0.85)
MMP7	1.85	1.20-2.84	1.72	1.35-2.19	0.714	0.97 (0.92-1.00)	0.98 (0.95-1.00)
PICP ^	1.06	0.95-1.18	1.03	0.97-1.10	0.631	0.53 (0.34-0.71)	0.56 (0.46-0.66)
PLG ^	1.12	1.03-1.21	1.08	1.02-1.14	0.362	0.71 (0.56-0.87)	0.65 (0.55-0.74)
TSP2 ^	1.35	1.09-1.69	1.37	1.18-1.59	0.915	0.69 (0.51-0.87)	0.78 (0.69-0.87)
IGFBP2IGFBP2	1.09	1.03-1.15	1.09	1.05-1.14	0.900	0.78 (0.62-0.94)	0.83 (0.76-0.90)
FN ^	0.97	0.89-1.06	0.96	0.92-1.01	0.833	0.61 (0.43-0.78)	0.64 (0.54-0.74)
PINP	0.99	0.87-1.12	0.99	0.91-1.07	0.957	0.55 (0.36-0.74)	0.51 (0.41-0.62)
CCN1 ^	0.99	0.90-1.09	1.03	0.98-1.08	0.384	0.57 (0.39-0.74)	0.53 (0.43-0.64)
CCN2 ^	1.33	1.10-1.60	1.41	1.22-1.64	0.483	0.83 (0.69-0.97)	0.86 (0.79-0.93)
Col4 ^^	1.14	0.92-1.41	1.08	0.94-1.23	0.574	0.61 (0.45-0.78)	0.55 (0.44-0.65)
CA19.9 ^	1.79	0.96-3.34	1.97	1.38-2.81	0.733	0.81 (0.64-0.97)	0.89 (0.83-0.96)
CA19.9 ^+MMP7	2.69	1.08-6.71	3.02	1.56-5.85	0.792	0.98 (0.93-1.00)	0.99 (0.99-1.00)
CA19.9 ^+CCN2	2.38	1.21-4.67	2.77	1.65-4.65	0.646	0.93 (0.86-1.00)	0.96 (0.92-1.00)

Stroma- related molecules	PDAC vs Pancreatitis						
	Odds Ratio				P-value for an interaction between stages	AUC (99%CI)	
	Stage IA-IB-IIA		Stage IIB				
	Point estimate	99%CI	Point estimate	99%CI		Stage IA-IB-IIA	Stage IIB
TIMP1	1.02	0.99-1.04	1.02	1.00-1.04	0.906	0.61 (0.39-0.83)	0.65 (0.51-0.78)
sICAM1 ^	1.00	0.97-1.03	1.00	0.98-1.02	0.311	0.45 (0.23-0.66)	0.56 (0.40-0.72)
MMP7	1.01	0.97-1.05	1.02	0.98-1.06	0.676	0.61 (0.40-0.82)	0.67 (0.52-0.81)
PICP ^	0.99	0.86-1.14	1.00	0.90-1.12	0.864	0.51 (0.28-0.73)	0.53 (0.38-0.67)
PLG ^	1.15	1.02-1.29	1.12	1.02-1.23	0.674	0.77 (0.60-0.94)	0.73 (0.59-0.86)
TSP2 ^	0.99	0.92-1.07	1.02	0.97-1.08	0.389	0.54 (0.34-0.75)	0.57 (0.42-0.73)
IGFBP2	1.01	0.98-1.04	1.01	0.99-1.03	0.776	0.53 (0.31-0.76)	0.59 (0.45-0.73)
FN ^	1.18	1.01-1.38	1.19	1.03-1.36	0.969	0.81 (0.64-0.97)	0.79 (0.65-0.94)
PINP	0.92	0.79-1.06	0.92	0.82-1.04	0.898	0.63 (0.43-0.84)	0.60 (0.45-0.76)
CCN1 ^	1.06	0.93-1.22	1.07	0.98-1.17	0.870	0.58 (0.37-0.80)	0.65 (0.51-0.78)
CCN2 ^	0.99	0.95-1.03	1.01	0.98-1.04	0.239	0.57 (0.36-0.80)	0.58 (0.43-0.73)
Col4 ^^	1.41	1.04-1.90	1.39	1.07-1.79	0.918	0.78 (0.60-0.95)	0.74 (0.59-0.89)
CA19.9 ^	1.15	0.99-1.35	1.19	0.99-1.43	0.728	0.76 (0.56-0.95)	0.85 (0.76-0.94)
CA19.9 ^+CCN2 +Col4+FN+PLG	2.28	1.17-4.42	2.87	1.49-5.56	0.522	0.92 (0.83-1.00)	0.93 (0.87-0.99)

A standard adjusted analysis was performed. Sex strata were combined
AUC was estimated non-parametrically
^10 units
^^100 units

Appendix Table S3. Biomarkers in PdxCre/LSL-Kras^{G12D} (KC) and Kras^{G12D}/Trp53^{R172H} (KPC) GEM models.

A Exact n and p-values of Figure 3A

KC MICE

	TIMP1		MMP7		TSP2	
Days	n	p *	n	p *	n	p *
60	7		7		8	
120	8	ns	8	ns	8	ns
180	8	ns	9	ns	9	ns
240	8	0.0093	8	0.0500	8	0.0148
330	7	0.0262	8	0.0289	8	0.0047

KPC MICE

	TIMP1		MMP7		TSP2	
Days	n	p **	n	p **	n	p **
30	7		7		5	
90	6	ns	8	ns	7	ns
150	4	0.0424	4	0.0424	3	0.0357

B Exact n of Figure 3B

Groups	TIMP1	MMP7	TSP2
Healthy	15	15	15
Chronic P.	19	19	19
PdxCre	3	4	4
KC	7	8	8
PdxCre	7	7	4
KPC	4	4	4

* compared to 60 days
**compared to 30 days

Appendix Table S4. Murine circulating TIMP1, MMP7 and TSP2 as biomarkers of treatment response in PDAC-PDX models.

A Exact n and p-values of Figure 4A

	TIMP1		MMP7		TSP2	
Groups	n	p *	n	p *	n	p *
Healthy	3		4		4	
HuPa4	5	0.0357	4	0.0159	4	0.0159
HuPa8	8	0.0121	4	0.0286	4	0.0286
HuPa11	2	0.0078	5	0.0286	5	0.0286

B Exact p-values of Figure 4C

	TIMP1	MMP7	THBS2	CCN2	ICAM1	IGFBP2	PLG
Groups	p *	p *	p *	p *	p *	p *	p *
HuPa4	0.0061	ns	0.0061	0.0061	0.0061	0.0242	ns
HuPa8	0.0061	ns	0.0061	0.0061	0.0061	ns	ns
HuPa11	0.0061	ns	0.0061	0.0061	0.0121	ns	ns

* compared to Healthy

Appendix Table S5. Murine circulating TIMP1, MMP7 and TSP2 as biomarkers of treatment response in PDAC-PDX models.

A Exact n and p-values of Figure 5A

	TIMP1		MMP7		TSP2	
Days	n	p *	n	p *	n	p *
Healthy	3		4		4	
30	2		4		2	
90	2		4	0.0286	4	0.0450
150	8	0.0121	4	0.0286	4	0.0286

B Exact n of Figure 5D

	TIMP1			MMP7			TSP2		
Treatments	Days			Days			Days		
	80	120	165	80	120	165	80	120	165
Vehicle	5	5	6	5	5	6	5	5	5
GEM	5	3	3	5	4	4	5	4	3
GEM+NAB-P	5	3	5	5	4	5	5	4	3

* compared to Healthy
