# Supplementary material for: Soluble stroma‐related biomarkers of pancreatic cancer
Source: EMBO Mol Med. 2018 Jun 25;10(8):e8741. doi: 10.15252/emmm.201708741 (PMC6079536; doi:10.15252/emmm.201708741)
Supplement: Supplementary file 2 — Expanded View Figures PDF [file EMMM-10-e8741-s002.pdf]

Expanded View Figures

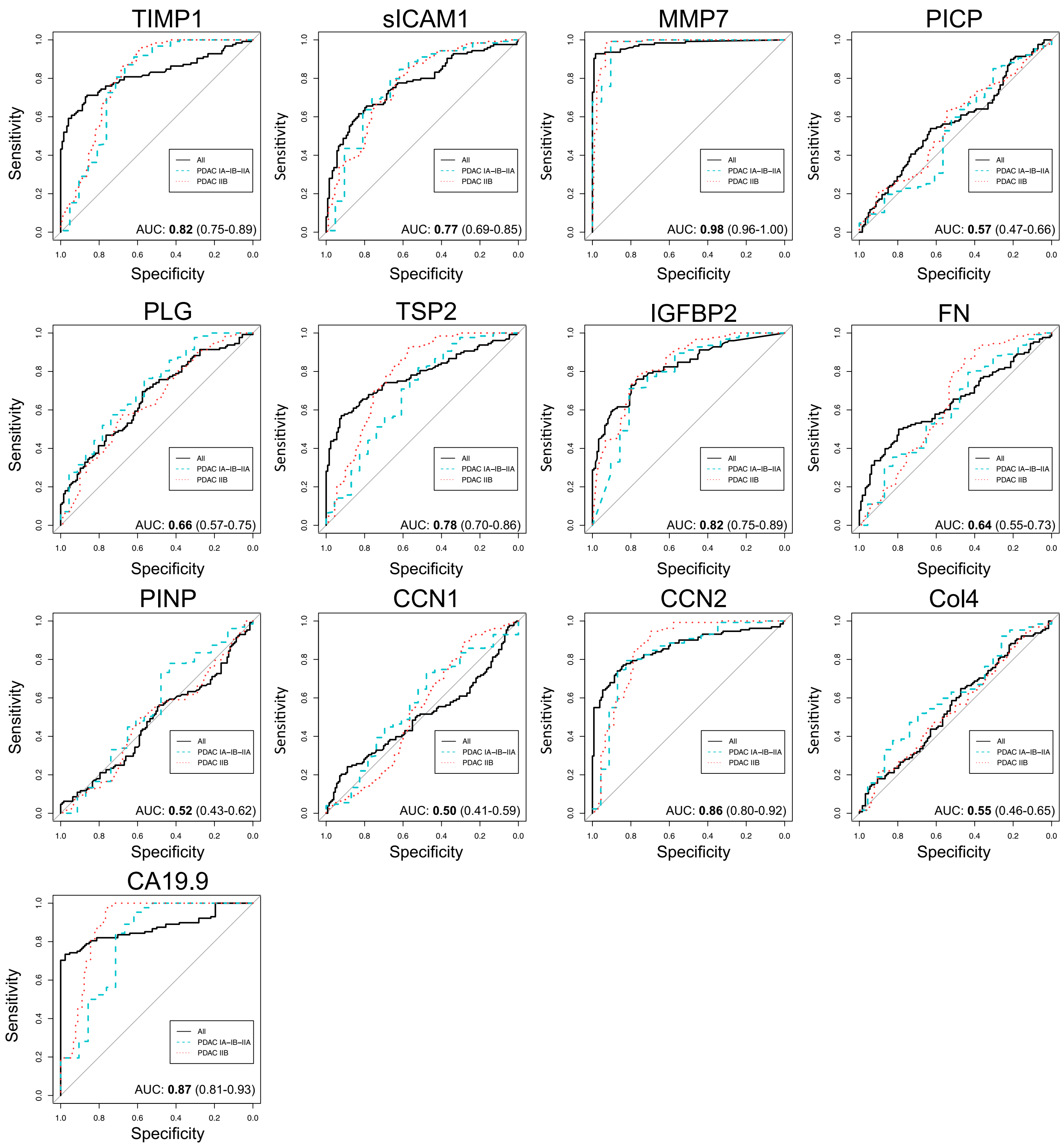

Figure EV1. Phase II confirmatory phase: AUC values of selected molecules in plasma of PDAC patients divided by stages.

Receiver operator characteristic (ROC) curves for diagnosis of PDAC versus healthy controls (not divided (All), early stages (IA, IB, and IIA), and late stages (IIB)). Areas under the curve (AUC) with 99% CI are presented for the whole population.

© 2018 The Authors
