# Supplementary material for: Soluble stroma‐related biomarkers of pancreatic cancer
Source: EMBO Mol Med. 2018 Jun 25;10(8):e8741. doi: 10.15252/emmm.201708741 (PMC6079536; doi:10.15252/emmm.201708741)
Supplement: Supplementary file 3 — Table EV1 [file EMMM-10-e8741-s003.docx]

| **Table EV1. Candidate biomarkers selected for analysis in stage I** | | | | |
| --- | --- | --- | --- | --- |
| EXTRACELLULAR MATRIX AND ADHESION MOLECULES |  | PICP |  | C-Terminal propeptide of Procollagene 1 |
|  |  | Col4 |  | Collagen 4 |
|  |  | CCN1/Cyr-61 |  | Cysteine-rich angiogenic inducer 61 |
|  |  | FN |  | Fibronectin |
|  |  | Lam-P1 |  | Laminin Proteolitic Fragment 1 |
|  |  | PINP |  | N-Terminal propeptide of Procollagene 1 |
|  |  | sICAM1 |  | Soluble Intercellular Adhesion Molecule 1 |
|  |  | sVCAM1 |  | Soluble Vascular Cell Adhesion Molecule 1 |
|  |  | SPARC |  | Secreted Protein Acidic and Rich in Cysteine |
|  |  | VTN |  | Vitronectin |
|  |  |  |  |  |
| PROTEASES |  | MMP2 |  | Matrix Metalloproteinase 2 |
|  |  | MMP3 |  | Matrix Metalloproteinase 3 |
|  |  | MMP7 |  | Matrix Metalloproteinase 7 |
|  |  | MMP9 |  | Matrix Metalloproteinase 9 |
|  |  | MMP12 |  | Matrix Metalloproteinase 12 |
|  |  | MMP13 |  | Matrix Metalloproteinase 13 |
|  |  | PLG |  | Plasminogen |
|  |  |  |  |  |
| PROTEASE INHIBITORS |  | α2M |  | alpha-2-Macroglobulin |
|  |  | TIMP1 |  | Metallopeptidase Inhibitor 1 |
|  |  | TIMP2 |  | Metallopeptidase Inhibitor 2 |
|  |  |  |  |  |
| ANTIANGIOGENIC FACTORS |  | ES |  | Endostatin |
|  |  | PEDF/SERPIN |  | Pigment Epithelium-Derived Factor |
|  |  | TSP1 |  | Thrombospondin 1 |
|  |  | TSP2 |  | Thrombospondin 2 |
|  |  |  |  |  |
| GROWTH FACTORS AND REGULATORS |  | CXCL7/NAP2 |  | Chemokine (C-X-C motif) Ligand 7 |
|  |  | CCN2/CTGF |  | Connective tissue growth factor |
|  |  | FGF-2 |  | Fibroblast Growth Factor 2 |
|  |  | IGFBP2 |  | Insulin-like Growth Factor-Binding Protein 2 |
|  |  | IGFBP4 |  | Insulin-like Growth Factor-Binding Protein 4 |
|  |  | IGFBP5 |  | Insulin-like Growth Factor-Binding Protein 5 |
|  |  | CCN3 |  | Nephroblastoma overexpressed |
|  |  | PDGF-BB |  | Platelet-Derived Growth Factor Subunit B |
|  |  | TGF-β1 |  | Trasforming Growth Factor Beta 1 |
|  |  | TGF-β2 |  | Trasforming Growth Factor Beta 2 |
|  |  | TGF-β3 |  | Trasforming Growth Factor Beta 3 |
|  |  | VEGFA |  | Vascular Endothelial Growth Factor A |
|  |  |  |  |  |
| OTHERS |  | NGAL/Lipocalin2 |  | Neutrophil Gelatinase-Associated Lipocalin 2 |
|  |  | PF4 |  | Platelet Factor 4 |
