# Supplementary material for: Soluble stroma‐related biomarkers of pancreatic cancer
Source: EMBO Mol Med. 2018 Jun 25;10(8):e8741. doi: 10.15252/emmm.201708741 (PMC6079536; doi:10.15252/emmm.201708741)
Supplement: Supplementary file 4 — Table EV2 [file EMMM-10-e8741-s004.docx]

| **Table EV2. Distribution of the 38 molecules analyzed in the screening phase.** | | | | | | | | | | | |
| --- | --- | --- | --- | --- | --- | --- | --- | --- | --- | --- | --- |
| **Stroma-related molecules** | **Unit** |  | **Healthy (16)** | | |  | **PDAC (25)** | | |  |  |
|  |  |  | **Mean** | **(Range)** | **S.D.** |  | **Mean** | **(Range)** | **S.D.** |  | **p-value**^(A)^ |
| MMP7 | ng/mL |  | 10.8 | (0.0-87.7) | 22.56 |  | 127.6 | (36.5-614.1) | 112.67 |  | 0.001 |
| Col4 | ng/mL |  | 251.9 | (187.3-367.2) | 49.29 |  | 955.3 | (232.9-4361.1) | 1297.92 |  | 0.002 |
| TIMP1 | ng/mL |  | 51.7 | (46.2-62.8) | 4.79 |  | 78.8 | (46.9-136.7) | 23.61 |  | 0.005 |
| TSP2 | ng/mL |  | 44.3 | (13.9-80.9) | 18.43 |  | 95.5 | (26.4-223.2) | 53.40 |  | 0.008 |
| CCN2 | pg/mL |  | 23.7 | (8.4-50.8) | 13.21 |  | 219.0 | (13.4-1399.9) | 313.95 |  | 0.009 |
| IGFBP2 | ng/mL |  | 12.7 | (3.4-36.8) | 9.50 |  | 39.8 | (3.9-124.9) | 33.30 |  | 0.014 |
| PLG | µg/mL |  | 135.8 | (98.5-197.9) | 27.21 |  | 161.2 | (71.9-316.2) | 47.91 |  | 0.054 |
| sICAM1 | ng/mL |  | 182.7 | (101.0-331.5) | 74.96 |  | 278.4 | (65.0-615.9) | 167.18 |  | 0.061 |
| ES | ng/mL |  | 121.0 | (90.4-166.1) | 16.29 |  | 199.1 | (61.0-520.0) | 134.23 |  | 0.064 |
| FN | µg/mL |  | 285.7 | (156.2-444.3) | 92.89 |  | 349.2 | (151.2-509.9) | 108.10 |  | 0.066 |
| PDGF-BB | ng/mL |  | 1.2 | (0.0-3.7) | 1.1 |  | 2.4 | (0.0-9.8) | 2.6 |  | 0.120 |
| PICP | ng/mL |  | 129.0 | (58.4-178.6) | 28.68 |  | 142.6 | (92.5-177.2) | 24.08 |  | 0.125 |
| PINP | ng/mL |  | 16.7 | (12.3-19.7) | 2.14 |  | 16.3 | (11.3-19.6) | 2.21 |  | 0.125 |
| sVCAM1 | µg/mL |  | 1.0 | (0.2-1.3) | 0.2 |  | 0.9 | (0.4-1.6) | 0.2 |  | 0.158 |
| IGFBP4 | ng/mL |  | 25.3 | (9.1-44.4) | 10.12 |  | 31.6 | (10.5-64.8) | 15.33 |  | 0.161 |
| SPARC | ng/mL |  | 72.3 | (21.3-138.6) | 35.72 |  | 100.3 | (20.2-318.0) | 78.51 |  | 0.202 |
| TGF-β3^(B)^ | pg/mL |  | 0.0 | (0.0-0.0) | 0.00 |  | 2.7 | (0.0-28.6) | 8.04 |  | 0.202^(C)^ |
| CCN1 | pg/mL |  | 150.2 | (109.4-196.8) | 24.76 |  | 162.4 | (120.1-257.8) | 32.57 |  | 0.211 |
| VEGFA | pg/mL |  | 64.2 | (0.0-323.7) | 108.55 |  | 203.4 | (0.0-1805.2) | 416.35 |  | 0.260 |
| PEDF/Serpin^(B)^ | µg/mL |  | 6.5 | (2.6-10.0) | 2.14 |  | 7.3 | (4.2-11.2) | 2.11 |  | 0.281 |
| TGF-β2^(B)^ | µg/mL |  | 0.9 | (0.4-2.0) | 0.4 |  | 1.3 | (0.4-5.2) | 1.2 |  | 0.324 |
| Lam-P1 | mU/mL |  | 213.2 | (98.8-458.5) | 123.98 |  | 296.3 | (62.1-1593.5) | 314.21 |  | 0.352 |
| MMP12 | ng/mL |  | 1.7 | (0.7-3.4) | 0.72 |  | 1.9 | (0.6-4.1) | 1.01 |  | 0.360 |
| MMP3^(B)^ | ng/mL |  | 5.5 | (1.5-13.3) | 4.02 |  | 4.5 | (1.8-13.2) | 2.90 |  | 0.383 |
| CCN3^(B)^ | ng/mL |  | 13.6 | (9.6-21.0) | 3.98 |  | 12.3 | (5.2-23.4) | 5.13 |  | 0.410 |
| CXCL7/NAP2^(B)^ | ng/mL |  | 333.9 | (109.7-519.2) | 120.39 |  | 297.7 | (35.8-695.9) | 177.71 |  | 0.491 |
| TGF-β1^(B)^ | ng/mL |  | 6.7 | (2.0-12.5) | 3.41 |  | 8.8 | (1.1-47.1) | 10.87 |  | 0.504 |
| PF4^(B)^ | µg/mL |  | 1.2 | (0.2-3.6) | 0.9 |  | 1.5 | (0.06-7.4) | 2.1 |  | 0.626 |
| MMP9^(B)^ | ng/mL |  | 49.6 | (28.5-86.9) | 19.96 |  | 54.0 | (22.5-170.7) | 31.12 |  | 0.635 |
| α2M^(B)^ | µg/mL |  | 392.0 | (246.0-623.1) | 96.45 |  | 380.1 | (284.4-600.4) | 65.85 |  | 0.646 |
| FGF-2 | pg/mL |  | 328.0 | (0.0-1033.7) | 397.79 |  | 424.8 | (0.0-2966.4) | 778.99 |  | 0.651 |
| TSP1^(B)^ | µg/mL |  | 4.7 | (1.2-9.9) | 2.2 |  | 5.2 | (0.2-20.0) | 5.2 |  | 0.709 |
| IGFBP5 | ng/mL |  | 46.8 | (11.0-80.8) | 18.86 |  | 49.9 | (0.0-121.5) | 32.21 |  | 0.717 |
| MMP2^(B)^ | ng/mL |  | 60.2 | (17.7-150.1) | 30.87 |  | 58.9 | (39.7-91.0) | 13.27 |  | 0.855 |
| TIMP2^(B)^ | ng/mL |  | 50.9 | (39.1-62.8) | 7.29 |  | 51.1 | (40.9-61.1) | 5.59 |  | 0.887 |
| NGAL | ng/mL |  | 90.5 | (24.0-130.2) | 24.16 |  | 89.8 | (55.5-185.5) | 28.36 |  | 0.942 |
| MMP13 | ng/mL |  | 261.8 | (114.1-502.4) | 98.47 |  | 258.5 | (0.0-766.7) | 293.32 |  | 0.958 |
| VTN^(B)^ | ng/mL |  | 161.3 | (91.0-263.5) | 42.81 |  | 160.2 | (102.2-214.4) | 28.38 |  | 0.961 |
| ^(A)^ p value from 1 d.f. Wald χ2 for association with outcome.  ^(B)^ Stroma-related molecules with more than 10% of missing values screened out from the variable reduction process.  ^(C)^ Because complete or quasi-complete separation of data points was detected the Wilcoxon rank-sum test was used instead of the Wald χ2 test. | | | | | | | | | | | |
|  | | | | | | | | | | | |
|  | | | | | | | | | | | |
