# Supplementary material for: Soluble stroma‐related biomarkers of pancreatic cancer
Source: EMBO Mol Med. 2018 Jun 25;10(8):e8741. doi: 10.15252/emmm.201708741 (PMC6079536; doi:10.15252/emmm.201708741)
Supplement: Supplementary file 5 — Table EV3 [file EMMM-10-e8741-s005.docx]

| **Table EV3. Distribution of the selected molecules analyzed in the confirmatory phase.** | | | | | | | | | | | | | |  |
| --- | --- | --- | --- | --- | --- | --- | --- | --- | --- | --- | --- | --- | --- | --- |
| **Stroma-related molecules** | **Unit** |  | **Healthy (131)** | | |  | **Pancreatitis (30)** | | |  | **PDAC (131)** | | | |
|  |  |  | **Mean** | **Range** | **S.D.** |  | **Mean** | **Range** | **S.D.** |  | **Mean** | **Range** | **S.D.** | |
| TIMP1 | ng/mL |  | 54.7 | 24.0-87.7 | 10.94 |  | 70.3 | 40.8-135.0 | 23.32 |  | 95.0 | 30.6-304.8 | 48.49 | |
| sICAM1 | ng/mL |  | 140.9 | 0.0-642.8 | 86.02 |  | 278.2 | 60.0-1914.8 | 336.69 |  | 311.4 | 0.0-1667.8 | 277.78 | |
| MMP7 | ng/mL |  | 2.6 | 0.0-14.3 | 3.00 |  | 19.9 | 0.7-106.8 | 19.98 |  | 26.8 | 0.0-120.3 | 20.26 | |
| PICP | ng/mL |  | 138.2 | 10.3-336.9 | 61.41 |  | 144.2 | 57.9-219.4 | 40.95 |  | 152.6 | 38.3-287.4 | 60.56 | |
| PLG | µg/mL |  | 200.0 | 88.2-365.5 | 63.84 |  | 179.5 | 70.6-302.5 | 61.95 |  | 250.6 | 80.5-883.5 | 100.61 | |
| TSP2 | ng/mL |  | 55.6 | 4.9-156.5 | 21.50 |  | 105.8 | 31.4-477.5 | 89.80 |  | 141.5 | 14.2-1021.4 | 150.85 | |
| IGFBP2 | ng/mL |  | 7.2 | 0.0-45.9 | 9.00 |  | 23.0 | 3.0-80.1 | 20.54 |  | 45.8 | 0.0-367.6 | 61.01 | |
| FN | µg/mL |  | 157.9 | 57.4-407.9 | 72.32 |  | 76.2 | 26.4-243.5 | 55.67 |  | 136.0 | 33.3-721.1 | 93.52 | |
| PINP | ng/mL |  | 16.3 | 5.3-25.0 | 4.38 |  | 17.8 | 8.1-26.0 | 4.56 |  | 16.0 | 4.8-28.8 | 4.93 | |
| CCN1 | pg/mL |  | 193.4 | 67.7-456.8 | 62.54 |  | 164.2 | 60.1-339.8 | 52.12 |  | 200.5 | 73.9-447.8 | 80.45 | |
| CCN2 | pg/mL |  | 35.7 | 6.9-201.4 | 26.20 |  | 152.3 | 7.1-891.4 | 182.94 |  | 229.9 | 8.5-2315.7 | 357.02 | |
| Col4 | ng/mL |  | 612.0 | 105.1-1382.7 | 260.24 |  | 448.5 | 179.4-1280.5 | 276.12 |  | 670.8 | 179.9-1434.7 | 281.06 | |
| CA19.9 | U/ml |  | 10.4 | 0.0-39.7 | 9.74 |  | 22.8 | 1.0-174.0 | 36.31 |  | 1576.6 | 2.0-21750.0 | 3399.35 | |
