# Supplementary material for: Soluble stroma‐related biomarkers of pancreatic cancer
Source: EMBO Mol Med. 2018 Jun 25;10(8):e8741. doi: 10.15252/emmm.201708741 (PMC6079536; doi:10.15252/emmm.201708741)
Supplement: Supplementary file 6 — Table EV4 [file EMMM-10-e8741-s006.docx]

| **Table EV4. Significance tests and AUC of the molecules analyzed in the confirmatory phase.** | | | | | | | | | | | | |
| --- | --- | --- | --- | --- | --- | --- | --- | --- | --- | --- | --- | --- |
| **Stroma- related molecules** | **PDAC vs Healthy** | | | | **Pancreatitis vs Healthy** | | | | **PDAC vs Pancreatitis** | | | |
|  | **Odds Ratio** | | **P-value** | **AUC (99%CI)** | **Odds Ratio** | | **P-value** | **AUC (99%CI)** | **Odds Ratio** | | **P-value** | **AUC (99%CI)** |
|  | **Point estimate** | **99%CI** |  |  | **Point estimate** | **99%CI** |  |  | **Point estimate** | **99%CI** |  |  |
| **TIMP1** | 1.08 | 1.05-1.11 | <0.001 | 0.82 (0.75-0.89) | 1.07 | 1.03-1.13 | <0.001 | 0.72 (0.57-0.87) | 1.02 | 1.00-1.04 | 0.026 | 0.66 (0.53-0.79) |
| **sICAM1 ^** | 1.09 | 1.05-1.14 | <0.001 | 0.77 (0.69-0.85) | 1.08 | 1.02-1.14 | <0.001 | 0.69 (0.53-0.85) | 1.00 | 0.98-1.02 | 0.829 | 0.57 (0.41-0.72) |
| **MMP7** | 1.75 | 1.39-2.21 | <0.001 | 0.98 (0.96-1.00) | 1.64 | 1.25-2.15 | <0.001 | 0.95 (0.89-1.00) | 1.02 | 0.98-1.06 | 0.209 | 0.65 (0.51-0.80) |
| **PICP ^** | 1.05 | 0.99-1.11 | 0.042 | 0.57 (0.47-0.66) | 1.07 | 0.97-1.18 | 0.084 | 0.54 (0.41-0.68) | 1.01 | 0.92-1.11 | 0.750 | 0.54 (0.41-0.67) |
| **PLG ^** | 1.09 | 1.04-1.14 | <0.001 | 0.66 (0.57-0.75) | 0.93 | 0.85-1.02 | 0.049 | 0.59 (0.44-0.74) | 1.13 | 1.03-1.24 | <0.001 | 0.74 (0.61-0.86) |
| **TSP2 ^** | 1.37 | 1.19-1.58 | <0.001 | 0.78 (0.70-0.86) | 1.46 | 1.17-1.83 | <0.001 | 0.72 (0.56-0.89) | 1.02 | 0.97-1.08 | 0.288 | 0.57 (0.42-0.72) |
| **IGFBP2** | 1.09 | 1.05-1.14 | <0.001 | 0.82 (0.75-0.89) | 1.09 | 1.04-1.15 | <0.001 | 0.81 (0.71-0.91) | 1.01 | 0.99-1.03 | 0.096 | 0.59 (0.45-0.72) |
| **FN ^** | 0.97 | 0.93-1.01 | 0.042 | 0.64 (0.55-0.73) | 0.71 | 0.60-0.85 | <0.001 | 0.86 (0.73-1.00) | 1.22 | 1.06-1.41 | <0.001 | 0.80 (0.65-0.94) |
| **PINP** | 0.99 | 0.92-1.06 | 0.611 | 0.52 (0.43-0.62) | 1.09 | 0.96-1.22 | 0.067 | 0.60 (0.44-0.75) | 0.93 | 0.83-1.04 | 0.082 | 0.61 (0.46-0.76) |
| **CCN1 ^** | 1.02 | 0.97-1.06 | 0.395 | 0.50 (0.41-0.59) | 0.93 | 0.84-1.03 | 0.047 | 0.67 (0.53-0.81) | 1.06 | 0.97-1.16 | 0.068 | 0.63 (0.50-0.75) |
| **CCN2 ^** | 1.41 | 1.24-1.62 | <0.001 | 0.86 (0.80-0.92) | 1.34 | 1.13-1.58 | <0.001 | 0.83 (0.69-0.96) | 1.01 | 0.98-1.04 | 0.193 | 0.56 (0.41-0.71) |
| **Col4 ^^** | 1.08 | 0.96-1.22 | 0.089 | 0.55 (0.46-0.65) | 0.74 | 0.58-0.95 | 0.001 | 0.70 (0.55-0.85) | 1.41 | 1.10-1.81 | <0.001 | 0.74 (0.59-0.89) |
| **CA19.9 ^** | 1.79 | 1.32-2.44 | <0.001 | 0.87 (0.81-0.93) | 1.42 | 0.96-2.12 | 0.022 | 0.60 (0.43-0.76) | 1.17 | 0.99-1.37 | 0.016 | 0.83 (0.75-0.92) |
| A standard adjusted analysis was performed. Sex strata were combined  Likelihood ratio test was used  AUC was estimated non-parametrically  ^10 units  ^^100 units | | | | | | | | | | | | |
|  | | | | | | | | | | | | |
|  | | | | | | | | | | | | |
|  | | | | | | | | | | | | |
